# Backwards-compatibility in GENESIS 3.0 and beyond: bridging between procedural and declarative modeling

**DOI:** 10.1186/1471-2202-12-S1-P17

**Published:** 2011-07-18

**Authors:** Hugo Cornelis, Allan C Coop, Armando L Rodriguez, David Beeman, James M Bower

**Affiliations:** 1 Department of Neurophysiology, Catholic University of Leuven, Leuven, 3000, Belgium; 2 Merindah Energy Ltd., Freemansreach, NSW, 2756, Australia; 3 Research Imaging Institute, University of Texas Health Science Center, San Antonio, TX 78229, USA; 4 Department of Electrical, Computer, and Energy Engineering, University of Colorado, Boulder, CO 80309, USA

## 

Neural models have traditionally been described using procedural scripts with loops and conditional statements. The user-friendliness of a procedural scripting paradigm allows researchers to easily combine the model with an appropriate experimental paradigm of choice. As a consequence most popular models have been encoded in script interpreters such as the GENESIS 2 script language (SLI) or NEURON hoc files. The modularity of these object-oriented and procedural simulator scripts makes it easy to reuse, expand and share them. However the joint expression of model and experimental paradigm in the same set of scripts also makes it difficult to isolate either model or experimental paradigm and reuse them in new research or collaborations. The model itself, embedded in an inaccessible procedural syntax, has become unavailable to external software components for further analysis, visualization or other forms of processing.

GENESIS 3 (G-3) is a backwards compatible and modular reimplementation of GENESIS 2. The G-3 framework not only preserves the advantages of procedural and object-oriented scripting, but combines them with the advantages of the declarative modeling features necessary to make both model and experimental paradigm accessible to external software components.

G-3 eliminates the need for much scripting through use of GUIs and a declarative representation in its native Neurospaces Description Format (NDF). The procedure to construct a single neuron model with appropriate stimulii is well understood and easily converted to a declarative representation. However, a procedural representation is often better suited for describing certain aspects of a model or its simulation environment. In network simulations, the specification of input and output can be as complicated as the model.

The challenge is to provide both the user-friendliness of a procedural description and the interfacing capability of a declarative description. This is achieved in G-3 with NDF, which is unique in its ability to store both declarative and algorithmic procedural descriptions of not only the model, but the entire experimental protocol of the simulation. This is done for existing GENESIS 2 scripts with the ns-sli module, which provides backwards compatibility between SLI scripts and the NDF representation.

Although the ns-sli module is a key component for the importation of existing GENESIS 2 models, we expect future scripting to be done with Python, using additional Python modules developed for G-3. These provide Python bindings for each component of the G-3 architecture. For example, SSPy provides a Python implementation of the scheduler used by G-3. Other powerful software components will be described during the workshop "Using models to collaborate, communicate and publish". These interoperable components fully comply with the user workflow for enhanced interfacing with databases, GUIs, and each other.

Because this design is based on software components with clearly identified functionality rather than on complete simulators that are "all-in-one boxes", the approach taken by G-3 broadens the definition of interoperability between simulators to that of interfacing between software components.

